# Dendrobine biosynthesis in *Dendrobium nobile* in four different habitats is affected by the variations in the endophytic fungal community

**DOI:** 10.3389/fmicb.2022.981070

**Published:** 2022-09-13

**Authors:** Lin Li, Chaobo Liu, Wei’e Wen, Qingqing Li, Tiantian Pan, Zhaogao Li, Gang Qian, Yuqi He, Delin Xu

**Affiliations:** ^1^Department of Cell Biology, Zunyi Medical University, Zunyi, Guizhou, China; ^2^School of Pharmacy, Zunyi Medical University, Zunyi, Guizhou, China; ^3^Engineering Research Center of Key Technology Development for Guizhou Provincial Dendrobium nobile Industry, Zunyi Medical University, Zunyi, Guizhou, China

**Keywords:** *Dendrobium nobile*, endophytic fungi, altitude, dendrobine, habitats

## Abstract

*Dendrobium nobile*, an epiphytic plant, is a traditional medicinal herb with abundant endophytes. It is unclear whether the variation in the diversity and abundance of endophytes could stimulate the biosynthesis of medicinal compounds in the plant. In this study, we collected fresh stems of *D. nobile* from four habitats for investigating the fungal community structure, dendrobine content, and environment factors and their correlations. The results indicated no significant difference in endophytic fungal diversity among the habitats; however, different dominant or special endophytic genera were observed in the hosts from different habitats. The altitude was observed to be positively related to the dendrobine content, as the stems collected from the altitude of 692 m exhibited the highest level of dendrobine. Furthermore, the relative abundance of *Toxicocladosporium* was found to be positively correlated with the altitude and dendrobine content. The epiphytic matrix exhibited a significant negative correlation with the relative abundance of the endophytic fungus *Gibberella* but did not exhibit any significant correlation with the dendrobine content. The results indicated that the abundance of endophytes in *D. nobile* was affected by the altitude and epiphytic matrix and that high *Toxicocladosporium* abundance and high altitude were conducive to dendrobine production.

## Introduction

*Dendrobium nobile* Lindl. is a perennial herb from the Orchidaceae family that is mainly distributed in Guizhou, Sichuan, Guangxi, and Yunnan Provinces in Southwest China (Adhikari et al., 2021), the climate of which usually is humid and hot. It is a well-known traditional Chinese herb and has been used for more than 1,500 years as a medicine in Asia (Nie et al., 2020). Pharmacological studies have confirmed its diverse effects including immunomodulatory, antitumor, antidiabetic, neuroprotective, and antioxidative (Teixeira da Silva and Ng, 2017; Li D. D. et al., 2022). Its chemical components such as polysaccharides and alkaloids (Li et al., 2020; Zhang et al., 2021) are the basis of its pharmacological action. However, several studies have reported that the chemical constituents of *D. nobile* vary across different habitats (Chen et al., 2014; Wang et al., 2021). For example, 3-hydroxy-3-methylglutaryl is present only in the samples from Yunnan (Wang et al., 2021). Moreover, many factors such as light intensity, water content, and microorganisms influence the abundance of chemical constituents in *D. nobile* (Li et al., 2017; Chen X. Y. et al., 2021). Hence, to better control the quality of *D. nobile* in planting, elucidating the effect of environmental factors on the synthesis of metabolites is necessary.

Endophytes are the microorganisms that are abundantly present in plant tissues and exert crucial effects on host plant growth, development, and adaptability (Jia et al., 2016; Li Q. et al., 2022). In plant micro-ecosystems, hundreds of endophytes function synergistically to assist the host plant in nutrient uptake and pathogen resistance (Nguyen et al., 2021). However, environmental factors have been reported to shape the endophytic fungal community structure, resulting in differences in plant characteristics (Coleman-Derr et al., 2016; Trivedi et al., 2020). Transplantation of plants from dam areas to mountains can lead to the recombination of endophytic fungi and alter the diversity and richness of endophytic fungi in *Ligusticum chuanxiong* (Kang et al., 2021). Interestingly, a study indicated that five secondary metabolites, namely aloe-emodin, rhein, emodin, chrysophanol, and physcion, are positively correlated with the diversity and abundance of endophytic fungi of *Rheum palmatum* from eight different production areas (Chen D. et al., 2021). Therefore, elucidating the community structure of endophytes can help reveal the role of endophytes in the formation of host plant characteristics and the production of key secondary metabolites.

The production and accumulation of dendrobine, an important index component and an active component of *D. nobile*, have been extensively studied. A study proved that the dominant endophytic fungi are closely related to dendrobine accumulation in *D. nobile* (Chen X. Y. et al., 2021). Another study reported that the dendrobine content in the seedling supplying with endophytic fungi was significantly increased along with an improved growth state (Yan et al., 2016). The endophytic mycorrhiza fungal strain MF 23 was reported to help in increasing the dendrobine content in *Dendrobium* (Chen X. Y. et al., 2021). Some endophytic fungi such as *Trichoderma longibrachiatum* (Sarsaiya et al., 2020) isolated from *D. nobile* were reported to synthesize dendrobine. Significant differences were observed in the dendrobine content in *D. nobile* across various habitats (Yang et al., 2020). Thus, analyzing the community structure of endophytic fungi and the correlation between the abundance of endophytic fungi and dendrobine content in *D. nobile* holds great significance. In this study, we investigated the community structure of endophytic fungi in *D. nobile* from four habitats and analyzed the correlation between the endophytic fungal community composition and dendrobine content. The purpose of this study was to elucidate the mechanism of formation of dendrobine by different endophytic fungi and facilitate dendrobine production.

## Materials and methods

### Sample collection

**FIGURE 1:**
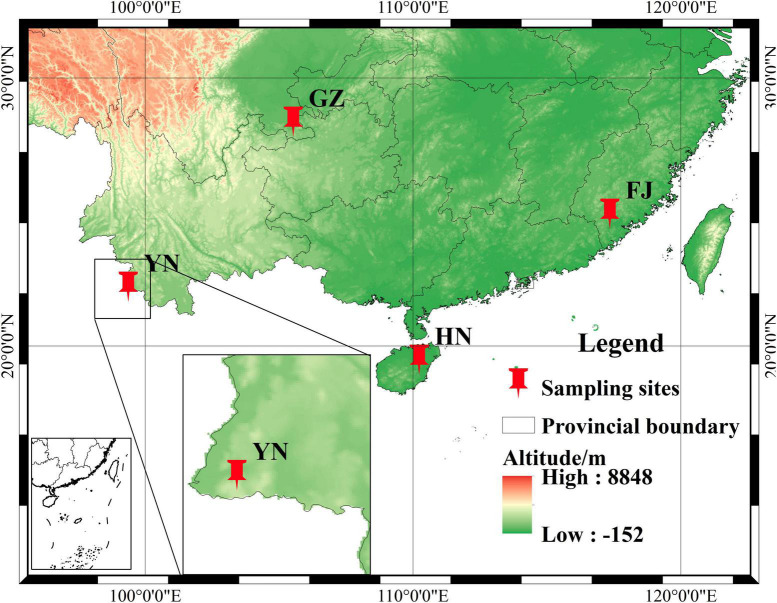
The geographical distribution map.

A total of 54 *D. nobile* stems were collected from four provinces (Figure 1), the number of which sampled from GZ, HN, YN, and FJ were 24, 24, 3, and 3, respectively. The stems of *D. nobile* that were growing on different substrates such as pine bark and stones were collected from Chishui City, Guizhou Province (GZ, N28°30′2″, E105°55′48″), Haikou city, Hainan Province (HN, N19°48′12″, E110°19′44″), Menglian county, Yunnan Province (YN, N22°20′8″, E99°37′6″), and Zhangzhou city, Fujian Province (FJ, N24°9′33″, E117°35′39″) of China. After removing the roots and leaves, the stems were packed in 50-mL centrifuge tubes, labeled. Then, all stems were disinfected as follows steps. Firstly, 20 ml 75% alcohol was added to 50 ml tube with 45 s. Secondly, after alcohol removed 20 ml 0.1% mercuric chloride was added for deepen disinfection with 5 min. Finally, sterile water was used to rinse the remaining mercuric chloride three times. All sterile stems were immediately stored at −80°C. Each sample was divided into two parts: one part with 200 mg stems of each *D. nobile* sample was sent to the Lianchuan Biological Company for internal transcribed spacer (ITS) sequencing, and the other part was used for determining the dendrobine content. The information of climate and growth environment factors of *D. nobile* are shown in Table 1.

**TABLE 1:** Information of all *Dendrobium nobile* samples.

Sample number	Collection places	Altitude (m)	Epiphytic substrate	Annual average temperature (°C)	Annual rainfall (mm)
YN18-20	Menglian	996.5	Pine bark	19.6	1373
GZ1-24	Chishui	327–692	Danxia stone	18.1	1292.3
FJ1,7,8	Zhangzhou	43	Pine bark	21	1500
HN1-6	Haikou	45–46	Areca catechu	23.8	1220.4
HN7-12	Haikou	44–54	Litchi chinensis	23.8	1220.4
HN13-18	Haikou	43–51	Jackfruit tree	23.8	1220.4
HN19-24	Haikou	22–48	Pelelith	23.8	1220.4

### DNA extraction, amplification, and sequencing

DNA from all stems was extracted using the E.Z.N.A. Stool DNA Kit (D4015, Omega, Inc, United States) according to the manufacturer’s instructions. Fresh stems (approximately 200 mg) were ground and transferred into 2.0-mL microcentrifuge tubes with 540 μL of SLX-Mlus buffer. To completely homogenized samples, 60 μL of DS buffer and 20 μL of proteinase K solution were added, and the mixture was incubated at 70°C for 10 min. Then, SP2 buffer was added, and the supernatant of the mixture was transferred to a collection tube for purification. Finally, the total DNA was eluted in 50 μL of elution buffer and stored at −80°C until performing PCR at LC-Bio Technology Co., Ltd., Hangzhou, Zhejiang Province, China.

The ITS2 region of the fungi was amplified with slightly modified versions of the primers ITS1FI2 (5′-GTGARTCATCGAATCTTTG-3′) and ITS2 (5′-TCCTCCGCTTATTGATATGC-3′) (Karlsson et al., 2014). PCR amplification was performed in a 25-μL reaction mixture containing 25 ng template DNA, 12.5 μL of PCR premix, and 2.5 μL of each primer, and PCR-grade water was used to adjust the volume of reaction mixture. The PCR conditions for amplification were as follows: an initial denaturation at 98°C for 30 s, followed by 32 cycles of denaturation at 98°C for 10 s; annealing at 54°C for 30 s; and extension at 72°C for 45 s, followed by a final extension at 72°C for 10 min. The PCR products were confirmed using 2% agarose gel electrophoresis, purified using AMPure XT beads (Beckman Coulter Genomics, Danvers, MA, United States), and quantified using Qubit (Invitrogen, United States). The amplicon pools were prepared for sequencing, and the size and quantity of the amplicon library were assessed using an Agilent 2100 Bioanalyzer (Agilent, United States) and Library Quantification Kit for Illumina (Kapa Biosciences, Woburn, MA, United States), respectively. The libraries were sequenced on the NovaSeq PE250 platform.

### Sequencing data analyses

The samples were sequenced on the Illumina NovaSeq platform according to the manufacture’s recommendations provided by LC-Bio. Paired-end reads were assigned based on their unique barcode and truncated by cutting off the barcode and primer sequence. Paired-end reads were merged using Pear. Quality filtering on the raw reads was performed under specific filtering conditions to obtain high-quality clean tags according to fqtrim software (version 0.94). Chimeric sequences were filtered using Vsearch software (version 2.3.4). After dereplication using DADA2, we obtained the feature table and feature sequences. Alpha diversity [observed operational taxonomic units (OTUs) and Shannon index] and beta diversity [principal coordinate analysis (PCoA) and non-metric multidimensional scaling (NMDS)] were calculated using QIIME2, in which the same number of sequences was randomly extracted by reducing the number of sequences of some samples to the minimum, and the relative abundance (X fungal count/total count) was used in fungus taxonomy. The sequence alignment of species annotation was performed using the QIIME2 plugin feature-classifier, and the alignment databases used were RDP and UNITE.

### Determination of the dendrobine content and data analysis

The clean stems were cut into small segments, dried at 60°C, and ground to powder. After sieving through No. 3 sieve, 0.25 mg powder of *D. nobile* was refluxed and extracted with 0.05% formic acid methanol solution (25 mL) according to the method described in Chinese Pharmacopoeia, 2020 (Chinese Pharmacopoeia Commission, 2020). This mixture was boiled in water at 80°C for 3 h and complemented weightlessness with methanol-0.05% formic acid solution after the mixture was cooled. Overall, 2 mL of filtered extract was transferred to a volumetric bottle containing 1 mL of internal standard stock solution (12.5 mg naphthalene dissolved in 10 mL of methanol), to which 5 mL methanol–0.05% formic acid solution was added for further detection. The dendrobine content was determined through gas chromatography (Lu et al., 2020); the chromatography system (Agilent 7820A, Flame ionization detector, Co., Agilent, United States) was equipped with a DB-1 GC column (column length, 30 m; internal diameter, 2.25 mm; and film thickness, 0.25 μm). The initial temperature was 80°C, which was increased to 250°C at a rate of 10°C/min and maintained for 5 min. The temperature of both sample inlet and detector was 250°C; the flow rate of the carrier gas was 1 mL/min, and the injection volume was 1 μL. The air flow rate was 300 mL/min, the hydrogen flow rate was 30 mL/min, the tail gas blowing rate was 25 mL/min, and the split ratio was 1:1.

Then, a differential analysis among different groups was performed *via t*-test in R language (version 4.0.5), *P*-value of < 0.05 was considered statistically significant.

### Correlation analysis of endophytic fungal abundance, dendrobine content, and habitat factors

The endophytic fungi in HN and GZ were sorted as per the relative abundance, and the top 5 genera were selected for further analysis. A correlation analysis was performed among endophytic fungal abundance, dendrobine content, and epiphytic matrix in HN. Moreover, the correlation analysis was performed among the endophytic fungal abundance, dendrobine content, and altitude for samples in GZ. The PerformanceAnalytics package in R language (version 4.0.5) and Spearman method were used for the correlation analysis. A *P*-value of < 0.05 was considered statistically significant.

## Results

### Sequence data results

**FIGURE 2:**
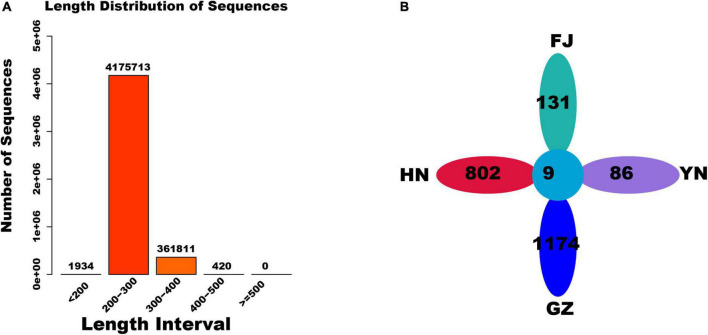
The sequence feature and length distribution of endophytic fungi. **(A)** Length distribution of sequences; **(B)** the number of feature sequences in four habitats.

After trimming the raw data of ITS2 sequencing, 4,539,878 reads were obtained from the *D. nobile* samples from four areas (Figure 2A). Most length of these reads was distributed in the 200–400 bp region. A total of 2,429 feature sequences were obtained after read denoising. A petal map analysis was further performed for all feature sequences. The results revealed that 9 feature sequences were shared by the *D. nobile* samples from four habitats, whereas the number of feature sequences unique to the HN, FJ, GZ, and YN habitats was 802, 131, 1174, and 86, respectively (Figure 2B).

### Diversity analysis of endophytic fungi

**FIGURE 3:**
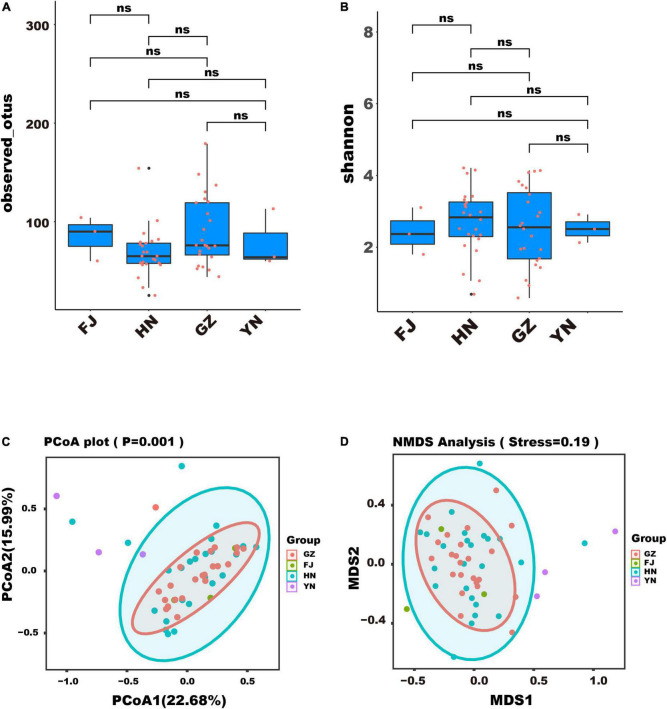
Diversity analysis of endophytic fungi of Dendrobium nobile from different habitats. **(A)** The index of observed OTUs; **(B)** the Shannon index; **(C)** the result of PCA; **(D)** the result of NMDS.

To assess the community composition of endophytic fungi in *D. nobile* in various habitats, we analyzed the diversity of endophytic fungi through alpha and beta analyses. The result of alpha analysis revealed no significant difference in the observed OTUs (Figure 3A) and Shannon index (Figure 3B), which indicated that the number and richness of endophytic fungi species in different habitats was similar. Moreover, the result of beta analysis showed that the community structure of endophytic fungi in different habitats could not be distinguished through PCA (Figure 3C) and NMDS (Figure 3D), implied that the community structure of endophytic fungi of *D. nobile* was similar across different habitats in this study.

### Comparison of the endophytic fungal community composition

**FIGURE 4:**
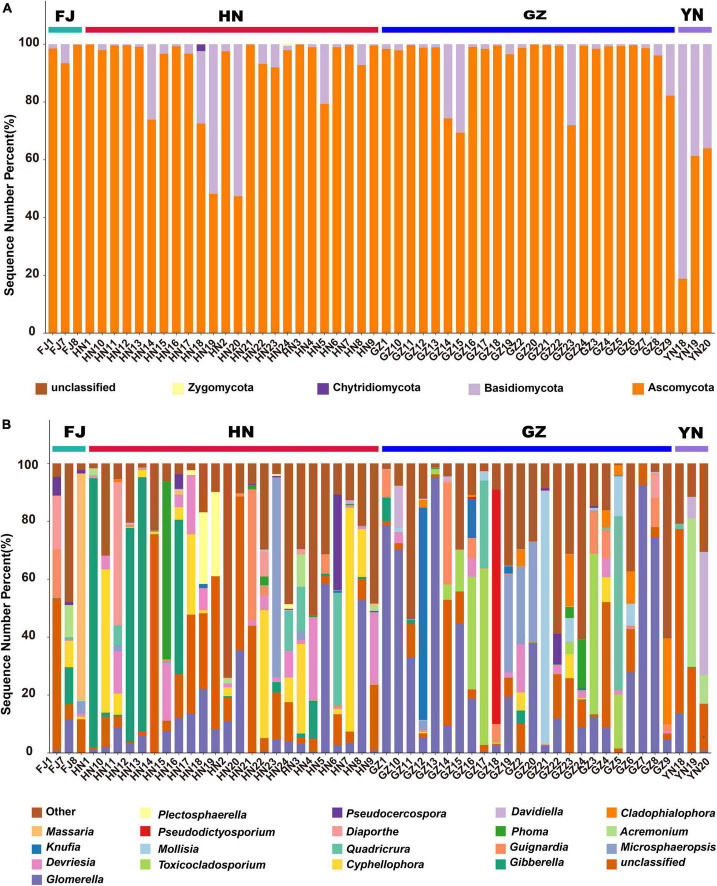
The endophytic fungal community composition of *Dendrobium nobile* from different habitats at the phylum **(A)** and genus **(B)** levels.

The annotation results revealed that all endophytic fungi belonged to four known phyla (Figure 4A), namely Ascomycota, Basidiomycota, Zygomycota, and Chytridiomycota. Among these phyla, Ascomycota was the main fungal flora with an average relative abundance of 82.75%. Notably, Chytridiomycota and Zygomycota were observed only in HN; however, their average relative abundance was extremely low (0.09 and 0.02%, respectively; Supplementary Table 1). At the genus level, all endophytic fungi were found to belonging 273 genera. Figure 4B shows the relative abundance of the top 20 genera. *Glomerella* (27.49%), *Acremonium* (20.40%), *Massaria* (26.03%) and *Gibberella* (13.78%), were the dominant endophytic fungi in *D. nobile* from GZ, YN, FJ, and HN, respectively (Supplementary Table 2). Except *Glomerella*, *Gibberella*, *Cyphellophora*, and Basidiomycota–unclassified, the *Devriesia* and *Toxicocladosporium* were among the top 5 genera with high relative abundance in HN and GZ, respectively; therefore, the correlation of the relative abundance of these genera with dendrobine content and environment factor was further analyzed.

### Comparison of the dendrobine content among *Dendrobium nobile* samples from different habitats

**FIGURE 5:**
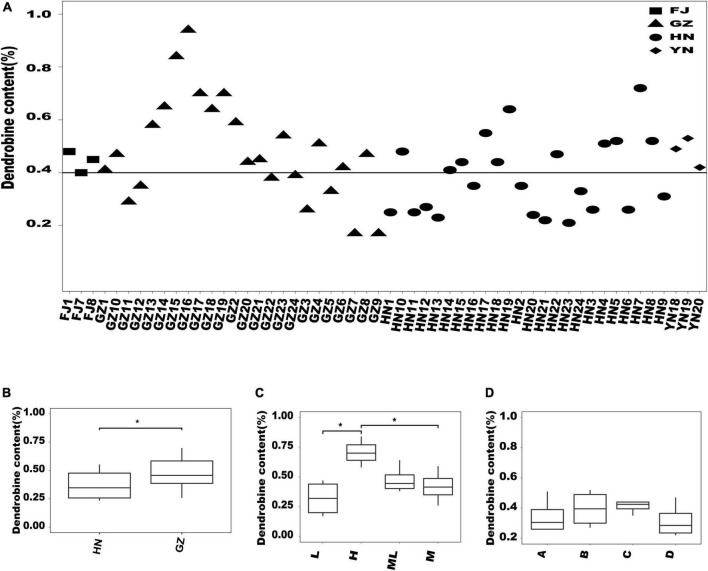
Analysis of the dendrobine content in *Dendrobium nobile* from different habitats. **(A)** Distribution of the dendrobine content of all samples; **(B)** comparison of the dendrobine content between HN and GZ; **(C)** comparison of the dendrobine content at different altitudes in GZ area. L, low altitude (327 m); H, high altitude (692 m); ML, middle-low altitude (484 m); M, middle altitude (528 m). *0.01 < *P* < 0.05; **(D)** comparison of dendrobine contents in different epiphytic substrates at the same altitude in HN producing area. A, areca catechu; B, litchi chinensis; C, jackfruit tree; and D, pelelith.

The dendrobine content varied among the *D. nobile* samples from different habitats. First, we found that among the 24 samples from GZ, the dendrobine content in 16 samples was >0.4%, which was higher than that in the samples from other habitats (Figure 5A). Then, the significant difference analysis between GZ and HN showed that the dendrobine content of *D. nobile* in GZ was significantly higher than that in HN (Figure 5B). In addition, we analyzed the difference in the dendrobine content according to the altitude in GZ (Figure 5C). The dendrobine content significantly varied between low and high altitudes and between middle and high altitudes. Further, we analyzed the difference in the dendrobine content in *D. nobile* growing on different substrates in HN; no significant difference in the dendrobine content was observed among *D. nobile* growing on different substrates at the same altitude (Figure 5D).

### Analysis of correlation among the dendrobine content and different habitat factors

**FIGURE 6:**
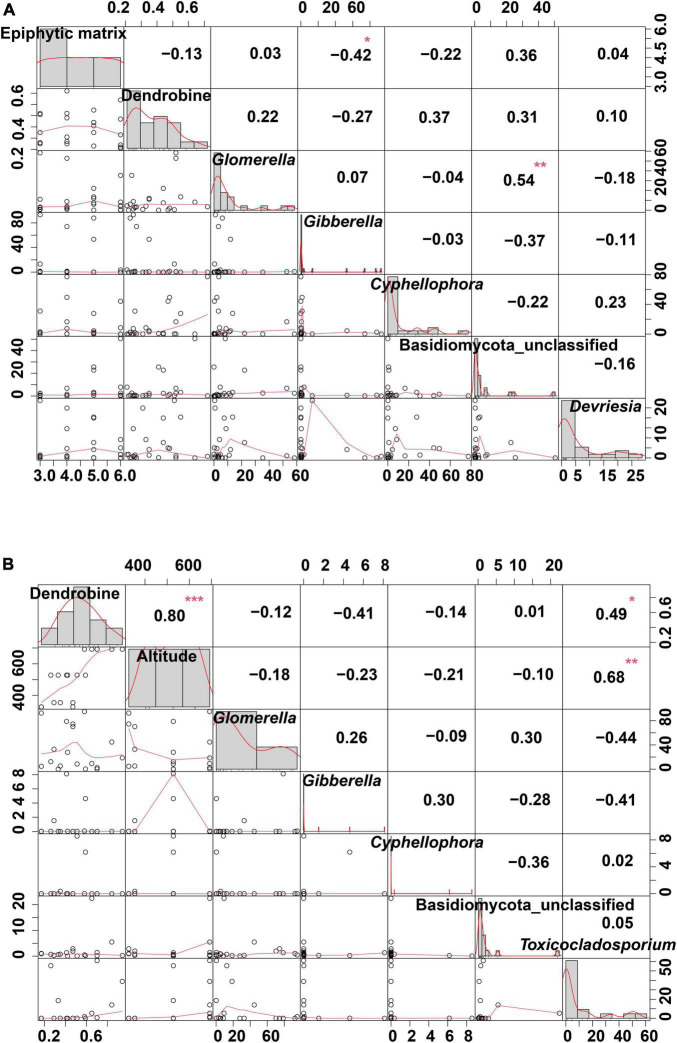
Correlation analysis of habitat factors and dendrobine content in *Dendrobium nobile*. **(A)** Correlation analysis among the dendrobine content, epiphytic matrix, and relative abundance of endophytic fungi in HN samples. **(B)** Correlation analysis among the dendrobine content, altitude, and relative abundance of endophytic fungi in *D. nobile* from GZ production area. *0.01 < *P* < 0.05; **0.001 < *P* < 0.01; and ***P < 0.001.

A correlation analysis was performed among the dendrobine content, endophytic fungi, and epiphytic matrix in *D. nobile* from HN (Figure 6A). The results revealed that the epiphytic matrix was very weakly correlated with the dendrobine content; however, the epiphytic matrix displayed a significant correlation with the relative abundance of *Gibberella* (*r* = −0.42, *P* < 0.05). Interestingly, Basidiomycota_classfied exhibited a significant correlation with the relative abundance of *Glomerella* (*r* = 0.54, *P* < 0.01). In addition, a correlation analysis was performed among the dendrobine content, altitude, and relative abundance of endophytic fungi in *D. nobile* from GZ (Figure 6B). The results revealed significant correlations between the dendrobine content and altitude (*r* = 0.80, *P* < 0.001), altitude and *Toxicocladosporium* abundance (*r* = 0.68, *P* < 0.01), and dendrobine content and *Toxicocladosporium* abundance (*r* = 0.49, *P* < 0.05).

## Discussion

The endophytic fungal community structure is closely related to the host plant genus and growth environment (Singer et al., 2019). In a certain area, the distribution of endophytes is relatively stable and exhibits differences in various plant species. Studies on plants such as sugarcane (De Souza et al., 2016) and citrus (Xu et al., 2018) have reported that a vast diversity of the fungi colonizing the plant tissues mainly belong to the phyla Ascomycota and Basidiomycota (Cregger et al., 2018; Hamonts et al., 2018). A study on five *Artemisia argyi* varieties in different cultivation areas reported significant differences in the dominant genera among the samples at the genus level (Wu et al., 2021). In this study, 2,429 characteristic sequences of endophytic fungi were obtained and annotated to four phyla, of which Ascomycota (90.41%) and Basidiomycota (9.53%) were found to be the most abundant in the four studied areas. The other two phyla, Chytridiomycota and Zygomycota, were identified only in HN. In addition, the species composition and relative abundance of the dominant genera of endophytic fungi varied across different producing areas. *Massaria* (26.03%), *Gibberella* (13.78%), *Glomerella* (27.49%), and *Acremonium* (20.40%) were the dominant endophytic fungi of *D. nobile* from FJ, HN, GZ, and YN habitats, respectively. However, the diversity of endophytic fungi of *D. nobile* from different habitats exhibited no significant difference, which indicates that the endophyte community of *D. nobile* cannot be significantly shaped by the habitats. The mechanism of formation of varied endophytic communities may be more closely correlated with other factors including the genetic factor.

Endophytes play a vital role in the regulation of synthesis pathways of chemical compounds (Yan et al., 2019). *Fusarium oxysporum* in *Passiflora incarnata* (da Silva et al., 2020), *Penicillium chrysogenum* in *Huperzia serrata* (Cao et al., 2021), and *Diaporthe* sp. in *Rhizophora stylosa* (Nagarajan et al., 2021) have been identified as the dominant species that promote the synthesis of active ingredients in host plants. Moreover, some endophytic fungi such as *Periconia* sp. (Ortega et al., 2021) and *Phoma* sp. (Kim et al., 2019) have been reported to produce new active substances, which has promoted the research on endophytes. In *Gentiana*, the loganic acid content was reported to be significantly positively correlated with the relative abundance of endophytic fungi (Hou et al., 2022). The liquiritin content of three *Glycyrrhiza* species was affected by the differences in the diversity of the endophytic fungal community (Dang et al., 2021). The accumulation of seven active components including total sugars, flavonoids, and ursolic acid in *Cynomorium songaricum* Rupr. was correlated with the change in the assembly of endophytic fungi with different plant developmental stages (Cui et al., 2018). In the present study, the relative abundance of fungi in *D. nobile* exhibited either a positive correlation or a negative correlation with the dendrobine content. *Gibberella* abundance in HN and GZ was strongly negatively correlated with the dendrobine content. At the same time, *Basidiomycota*_classfied and *Cyphellophora* abundances in HN and *Toxicocadosporium* abundance in GZ exhibited a positive correlation with the dendrobine content. Moreover, same fungal genera in different areas possibly exhibited different correlations with chemical compound accumulation. *Glomerella* and *Cyphellophora* abundances exhibited a negative correlation with the dendrobine content in GZ, but a positive correlation with the dendrobine content in HN.

In other plant species, the altitude factor accounted for the differences in the relative abundance of endophytes. For example, *Hypogymnia hypotrypa* within lichen-associated fungal species was significantly affected by altitude at the phylum and class levels (Wang et al., 2016). Yuan et al. (2018) reported that the diversity of fungal communities in tobacco tended to decrease with an increase in the altitude. The dendrobine content in *D. nobile* at a high altitude was significantly higher than that at a low altitude (Lu et al., 2020). In our study, the dendrobine content varied among stems from four habitats with different altitudes. The dendrobine content in the samples from GZ was significantly positively affected by the altitude and relative abundance of *Toxicocadosporium*. This endophytic fungus was detected only in GZ, and its varied abundance further clarified the positive relationship with the altitude factor. The finding indicated that the relative abundance of the endophytic fungus *Toxicocladosporium* may be shaped by the altitude and related to the differences in the accumulated dendrobine content at different altitudes. However, the relationship among *Toxicocladosporium* abundance, altitude, and dendrobine content remains to be further studied.

In summary, the environmental factors related to the *D. nobile* growth can impact the endophytic fungal community. Through correlation analyses, we report that the interactions between the factors such as abundance of core fungi, altitude, and epiphytic matrix significantly affected the dendrobine production in *D. nobile*.

## Conclusion

In this study, we compared the endophytic fungal community of *D. nobile* from four habitats through ITS sequencing and analyzed the correlation among the relative abundance of endophytes, several environmental factors, and dendrobine content. The diversity of endophytic fungi did not vary across different habitats; however, the structural composition of endophytes at the phylum and genus levels was found to vary greatly. The change in the altitude and relative abundance of *Toxicocladosporium* in GZ significantly correlated with the dendrobine content. Moreover, the change in the epiphytic matrix could lead to variations in the relative abundance of endophytic fungi in HN. The study findings enhanced our understanding of the changes in the fungal community and chemicals under the influence of environmental factors. Moreover, our findings be useful in developing a strategy to grow high-quality *D. nobile* based on the altitude, abundance of endophytic fungi, and epiphytic matrix.

## Data availability statement

The data presented in the study are deposited in the NCBI repository, accession number PRJNA862722.

## Author contributions

DX, YH, and GQ conceived, supervised, and writing-reviewed the manuscript, designed the experiments, and cofounded and co-administrated the project. LL and CL originally wrote and writing-reviewed the draft. LL, CL, WW, QL, TP, and ZL performed the experiments and carried out the analysis. All authors approved the final version.
